# Structural Analysis of an Inulin-Type Fructan from *Ophiopogon japonicus* and Its Immunomodulatory Properties

**DOI:** 10.3390/molecules31183194

**Published:** 2026-09-10

**Authors:** Henan Sun, Hao Yu, Huiqiang Yu, Gaowa Saren, Ke Feng, Wenzhong Hu

**Affiliations:** 1 School of Life Sciences, Zhuhai College of Science and Technology, Zhuhai 519041, China; 2 College of Life Sciences, Jilin University, Changchun 130021, China; 3 Medical Sciences Division, Macau University of Science and Technology, Macao 999078, China

**Keywords:** *Ophiopogon japonicus*, inulin-type fructan, immunomodulatory activity, macrophage activation, NF-κB

## Abstract

A water-soluble, low-molecular-weight polysaccharide fraction, designated OJP-2, was extracted from the roots of *Ophiopogon japonicus*; its structural characteristics and immunomodulatory activity in macrophages were subsequently investigated. OJP-2 exhibited a narrow apparent molecular weight distribution with an average molecular weight (Mw) of 3568 Da and was primarily composed of fructose (0.784) and glucose (0.208). Structural analysis—integrating UV spectroscopy, Fourier-transform infrared (FT-IR) spectroscopy, and 1D/2D nuclear magnetic resonance (NMR) spectroscopy—revealed that OJP-2 is an inulin-type fructan. Its structure is characterized predominantly by β-(2→1)-linked Fruf chains and terminal α-D-Glcp residues, with potential signals indicating C-6-substituted Fruf units. In vitro immunological studies demonstrated that OJP-2 promoted nitric oxide (NO) production and enhanced the secretion of IL-6, IL-1β, and TNF-α in RAW264.7 macrophages. Under LPS/IFN-γ stimulation, OJP-2 induced non-monotonic changes in the CD86/CD206 macrophage phenotype, with the most pronounced effects observed at a concentration of 50 μg/mL. Furthermore, Western blot analysis showed that, compared to the Model group, treatment with OJP-2 resulted in a downward trend in the relative levels of p-p65/p65 and p-IκBα/IκBα across the tested concentration range. Collectively, these findings indicate that OJP-2 modulates macrophage activation phenotypes and influences NF-κB-related signaling pathways. Thus, OJP-2 is a candidate fructan for further investigation of immunomodulatory activity.

## 1. Introduction

*Ophiopogon japonicus* (Thunb.) Ker Gawl. is used as both a medicinal material and a food-related botanical resource in East Asia. Carbohydrates are among its major functional constituents, and purified polysaccharide or fructan fractions have been associated with antioxidant, metabolic, and immunological activities [1,2,3,4,5,6]. These materials are chemically diverse. Reported *O. japonicus* carbohydrates differ in extraction route, chromatographic behavior, apparent molecular mass, monosaccharide composition, glycosidic linkage, and degree of branching [1,2,3,4,5]. Consequently, a fraction name alone is insufficient to define its chemical identity or biological behavior. Linking a cellular response to a specific preparation first requires fraction-level purification and complementary structural characterization.

Fructans are fructose-based carbohydrates that vary widely in chain length and topology. Inulin-type fructans contain a backbone composed mainly of β-(2→1)-linked D-fructofuranosyl (Fruf) residues and frequently carry an α-D-glucopyranosyl (Glcp) residue at one terminus [7,8,9,10,11,12]. Linear chains may be accompanied by terminal Fruf residues or limited substitution at fructosyl C-6, producing structural variation within the same broad fructan class. Molecular size, terminal-residue composition, branching, hydration, and aggregation can influence solubility and interactions with biological systems [8,9,10]. Inulin-type fructans from several plant sources have also altered macrophage-associated and cytokine responses [13,14,15,16]. A combined analytical strategy is therefore needed: size-exclusion chromatography defines the apparent molecular mass distribution, monosaccharide analysis establishes the dominant sugar composition, and one- and two-dimensional NMR spectroscopy resolves residue and linkage features.

Preparation conditions are another source of carbohydrate diversity. Enzyme-assisted treatment can facilitate release of polysaccharides by disrupting carbohydrate-protein or cell-wall associations under relatively mild conditions. Microwave irradiation promotes rapid dielectric heating, whereas ultrasound produces cavitation, shear, and enhanced transfer between the plant matrix and solvent [17,18]. Ultrasound-assisted enzymatic or ultrasound-assisted extraction has been applied to polysaccharides from *Dendrobium*, pomelo, jujube, *Allium*, and lychee matrices [19,20,21,22,23]. Other studies have coupled ultrasound-based preparation with chromatographic purification, structural analysis, and evaluation of immunological or anti-inflammatory activity [24,25,26,27]. Simultaneous microwave–ultrasound treatment further combines rapid energy input with acoustic disruption and has been incorporated into integrated polysaccharide preparation workflows [28]. Papain pretreatment followed by concurrent microwave and ultrasound processing was selected here to prepare the crude *O. japonicus* carbohydrate material before anion-exchange and gel-filtration fractionation.

Inulin-type fructans and related botanical polysaccharides can influence macrophage metabolism, lysosomal activity, nitric-oxide-related readouts, and cytokine release [6,13,14,15,16]. These responses depend on the initial state of the macrophage. A carbohydrate may enhance selected functions in unstimulated cells yet attenuate signaling events in cells exposed to an inflammatory stimulus. This distinction is especially relevant to NF-κB, in which phosphorylation of p65 and IκBα accompanies activation of inflammatory transcriptional programs. Surface-marker changes also require a continuum-based interpretation because CD86 and CD206 can coexist across heterogeneous macrophage states rather than defining mutually exclusive M1 and M2 populations. Assessing basal functional readouts, stimulus-conditioned surface markers, and intracellular phosphorylation in parallel can therefore provide a more informative view of immunomodulation than any single endpoint.

The present study focused on OJP-2, a low-molecular-weight fraction obtained from *O. japonicus* roots by papain-assisted simultaneous microwave–ultrasound treatment followed by DEAE-52 cellulose and Sephadex G-100 chromatography. OJP-2 was characterized by HPGPC, HPAEC-ED, UV–visible and FT-IR spectroscopy, SEM, AFM, and one- and two-dimensional NMR spectroscopy. Its effects in RAW264.7 macrophages were then examined at three levels: basal metabolic, lysosomal, NO-related, and cytokine responses; CD86/CD206 distributions under LPS/IFN-γ conditioning; and NF-κB-related p65 and IκBα phosphorylation. This design was used to connect the chemical profile of a defined *O. japonicus* fructan fraction with concentration- and assay-context-dependent macrophage responses.

## 2. Results

### 2.1. Preparation, Fractionation, and Spectroscopic Features of OJP-2

**Figure 1:**
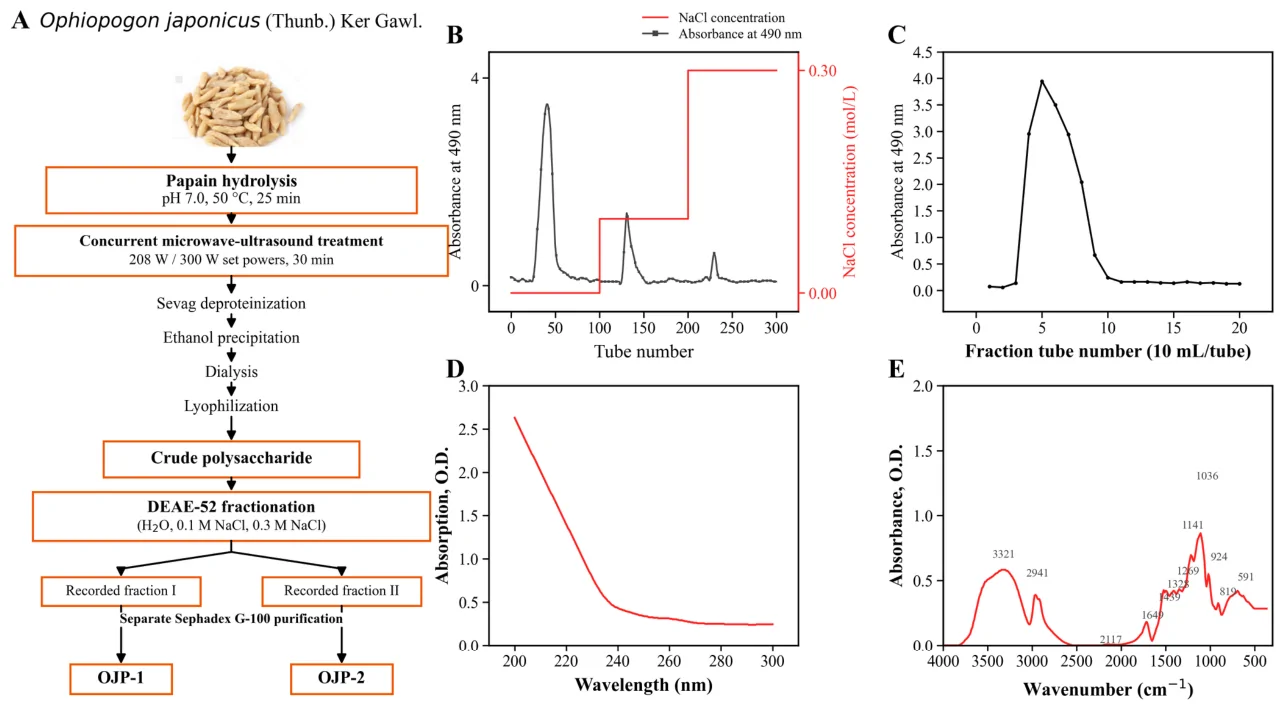
Preparation, fractionation, and spectroscopic characterization of OJP-2. (**A**) Papain-assisted simultaneous microwave–ultrasound preparation and two-stage chromatographic purification. Microwave irradiation (208 W) and ultrasound (300 W) were applied concurrently for 30 min. (**B**) DEAE-52 elution profile obtained with water, 0.1 M NaCl, and 0.3 M NaCl. (**C**) Sephadex G-100 elution profile of OJP-2. (**D**) UV–visible spectrum over 200–300 nm. (**E**) FT-IR spectrum.

Papain-assisted simultaneous microwave–ultrasound treatment followed by stepwise DEAE-52 elution and Sephadex G-100 purification yielded the OJP-1 and OJP-2 fractions (Figure 1A–C). The DEAE-52 profile contained several carbohydrate-positive features across the water, 0.1 M NaCl, and 0.3 M NaCl elution steps, and the second-stage chromatogram showed the purified OJP-2 fraction. Under the optimized settings (208 W microwave, 300 W ultrasound, 25 min enzymatic treatment, and 30 min extraction), five independent extractions gave crude-extract yields of 43.67%, 43.39%, 43.77%, 43.81%, and 43.20% (mean 43.568% ± 0.263%, n = 5; CV = 0.604%). This gravimetric value represents the dried 80% ethanol precipitate before chromatographic purification and was not corrected for total sugar purity. The five crude-extract yields and their calculation are presented in Supporting Information S7.

The sequential profiles document two complementary separation steps. DEAE-52 resolved the crude extract according to its interaction with the anion-exchange matrix, while Sephadex G-100 further separated the selected fraction according to hydrodynamic size. OJP-2 was the Sephadex G-100-purified product derived from the second DEAE-52 fraction and was carried forward as a single operational fraction for compositional, spectroscopic, microscopic, and cellular analyses.

The UV–visible spectrum showed no distinct absorption maximum at 280 nm; an extremely weak feature was discernible around 260 nm, while no peak-like increase in absorption was observed below 240 nm (Figure 1D). In the FT-IR spectrum (Figure 1E), bands occurred at approximately 3321, 2941, 1649, 1439, 1328, 1141, 1036, 924, 819, and 591 cm^−1^. The broad band at 3321 cm^−1^ was assigned to O–H stretching vibrations in hydroxyl groups and water molecules, and the signal at 2941 cm^−1^ to aliphatic C–H stretching. The accompanying band at 1649 cm^−1^ was attributed mainly to water/OH-associated bending vibrations, consistent with the lack of a pronounced carbonyl-related UV feature. The band at 1439 cm^−1^ was assigned to C–H bending vibrations. The 1328 cm^−1^ band lies in a carbohydrate region where overlapping C–C and C–O stretching and XCH bending vibrations can contribute. Signals at 1141 and 1036 cm^−1^ were characteristic of carbohydrate C–O and C–O–C vibrations, while the bands at 924 and 819 cm^−1^ were consistent with fructan/furanose features [29].

The strong O–H and carbohydrate fingerprint regions were consistent with a hydroxyl-rich carbohydrate material. The combination of the 1141 and 1036 cm^−1^ signals with the lower-wavenumber fructan-associated bands provided spectroscopic support for the chromatographic and monosaccharide results, although the detailed linkage assignment was derived from NMR rather than FT-IR alone.

### 2.2. Molecular-Mass Distribution, Monosaccharide Composition, and Morphology

**Figure 2:**
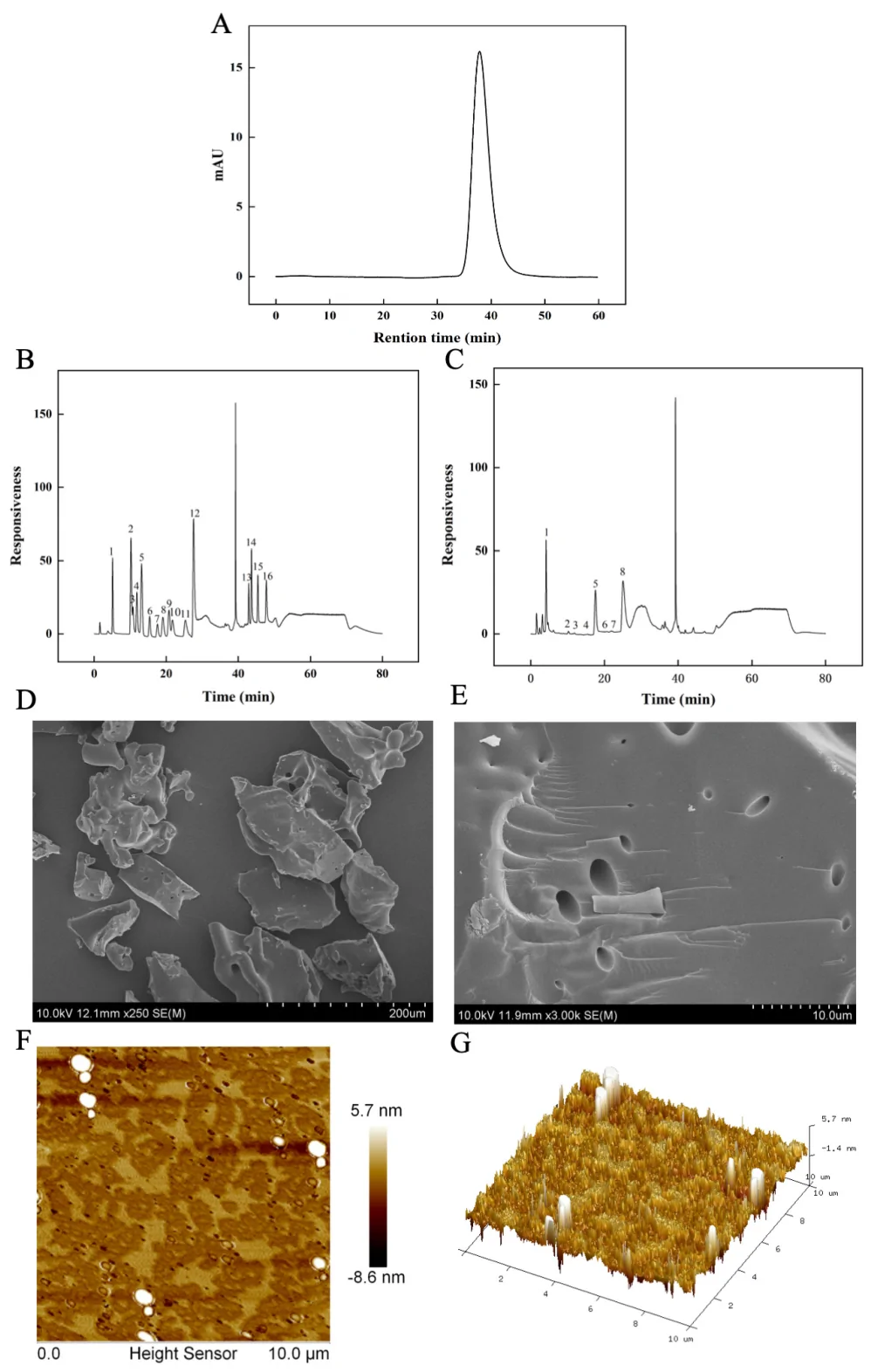
Molecular-mass distribution, monosaccharide composition, and morphology of OJP-2. (**A**) HPGPC trace and molecular-mass parameters. (**B**) HPAEC-ED chromatogram of 16 monosaccharide standards. (**C**) HPAEC-ED chromatogram of hydrolyzed OJP-2. (**D**,**E**) SEM images at 250× and 3000× (scale bars, 200 and 10 µm). (**F**,**G**) Two- and three-dimensional AFM views of OJP-2 deposited on mica over a 10 µm × 10 µm field. In (**B**), peaks 1–16 correspond to fucose, galactosamine, rhamnose, arabinose, glucosamine, galactose, glucose, N-acetylglucosamine, xylose, mannose, fructose, ribose, galacturonic acid, guluronic acid, glucuronic acid, and mannuronic acid, respectively. In (**C**), peaks 2–8 correspond to galactosamine, arabinose, galactose, glucose, xylose, mannose, and fructose, respectively; peak 1 denotes an unassigned early-eluting signal.

HPGPC analysis of OJP-2 produced a single narrow peak (Figure 2A). The calculated molecular-mass parameters were *M*_p_ = 3588 Da, *M*_w_ = 3568 Da, and *M*_n_ = 3553 Da, corresponding to *M*_w_/*M*_n_ = 1.004. OJP-2 therefore exhibited a narrow apparent molecular-mass distribution within the HPGPC detection range [30,31].

HPAEC-ED identified fructose and glucose as the dominant monosaccharides, with normalized molar fractions of 0.784 and 0.208, respectively (Figure 2B,C). Mannose accounted for 0.004, while galactosamine, arabinose, galactose, and xylose each accounted for 0.001. These data identify OJP-2 as a fructose-rich carbohydrate fraction.

The measured fructose-to-glucose ratio was approximately 3.77:1. Fructose predominance, together with glucose as the only substantial secondary monosaccharide, supported the fructose-rich classification, while the terminal α-D-Glcp assignment was derived from the NMR correlations. The trace monosaccharide signals together represented less than 1% of the normalized profile and did not alter this classification.

SEM images showed irregular sheet-like particles with pores and fragmented edges (Figure 2D,E).

AFM showed heterogeneous granular and sheet-like deposits across the 10 µm × 10 µm field (Figure 2F,G). The surface-height range extended from approximately −8.6 to 5.7 nm, with local protrusions and depressions.

Thus, the chromatographic analyses described OJP-2 as a narrowly distributed, low-molecular-weight fraction, whereas microscopy showed heterogeneous prepared-state particles and deposits in the lyophilized material after drying. These morphologies were larger-scale features and were considered separately from the molecular-mass distribution measured in solution.

### 2.3. NMR Features of OJP-2

**Figure 3:**
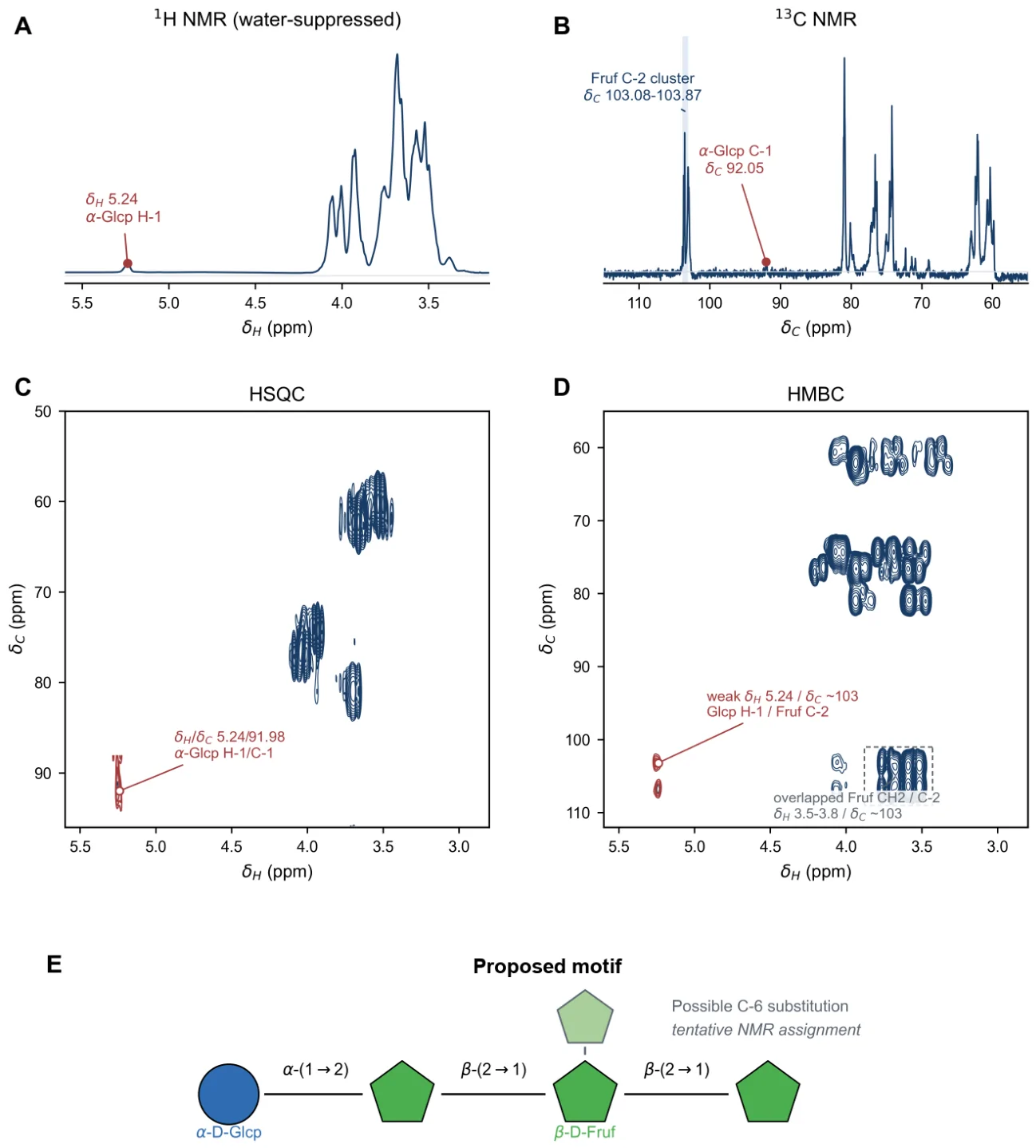
NMR spectra and proposed fructan motif of OJP-2. (**A**) Water-suppressed ^1^H NMR spectrum. (**B**) ^13^C NMR spectrum. (**C**) HSQC spectrum showing the α-D-Glcp H-1/C-1 cross-peak at δH/δC 5.24/91.98 ppm. (**D**) HMBC spectrum showing correlations from Glcp H-1 and Fruf methylene protons to the Fruf C-2 region. (**E**) Proposed inulin-type fructan motif containing an α-D-Glcp-(1→2)-β-D-Fruf end group, a predominantly β-(2→1)-linked Fruf sequence, and possible C-6 substitution. The C-6 feature is a tentative NMR assignment; the degree of polymerization, exact branch topology, and branch frequency were not determined.

The ^1^H spectrum was dominated by overlapping carbohydrate resonances at δ_H_ 3.3–4.1 ppm, together with a weak signal at δ_H_ 5.24 ppm and residual HDO at δ_H_ 4.64 ppm (Figure 3A). The principal ^13^C signals occurred at δ_C_ 59.82–81.06 ppm, with a weaker signal at δ_C_ 92.05 ppm and a cluster at δ_C_ 103.08–103.87 ppm (Figure 3B). The HSQC cross-peak at δ_H_/δ_C_ 5.24/91.98 ppm was assigned to H-1/C-1 of terminal α-D-Glcp, while the non-protonated signals at δ_C_ 103–104 ppm were assigned to the quaternary C-2 positions of β-D-Fruf residues.

Combined ^1^H, ^13^C, COSY, HSQC, HMBC, NOESY, and TOCSY analysis supported four residue classes in OJP-2 (Table 1).

HMBC showed a weak cross-peak near δ_H_/δ_C_ 5.24/~103 ppm and stronger correlations at δ_H_ 3.5–3.8/δ_C_ ~103 ppm. These features supported long-range contacts from Glcp H-1 and Fruf methylene protons to Fruf C-2, consistent with an α-D-Glcp-(1→2)-β-D-Fruf end group and a predominantly β-(2→1)-linked fructan chain. A separate weak signal class was consistent with possible C-6 substitution of Fruf, but this assignment remained tentative.

Together, the one- and two-dimensional NMR spectra supported an inulin-type fructan dominated by β-(2→1)-linked Fruf residues and containing a terminal α-D-Glcp unit [11,12,16]. The tentative C-6 substitution assignment was compatible with limited branching; however, the degree of polymerization, exact branch topology, and branch frequency were not determined. Figure 3E therefore presents a proposed structural motif, with the C-6 assignment marked as tentative.

The NMR interpretation was also internally consistent with the HPAEC-ED profile. The abundant Fruf signals accounted for the dominant fructose component, while the weaker terminal α-D-Glcp signal corresponded to glucose as the secondary monosaccharide. Agreement among the quaternary Fruf C-2 region, the Glcp anomeric correlation, and the long-range HMBC contacts distinguished OJP-2 from a generic glucose-rich polysaccharide and supported its assignment to the inulin-type fructan class.

### 2.4. OJP-2 Modulates Basal RAW264.7 Cell Activity

**Figure 4:**
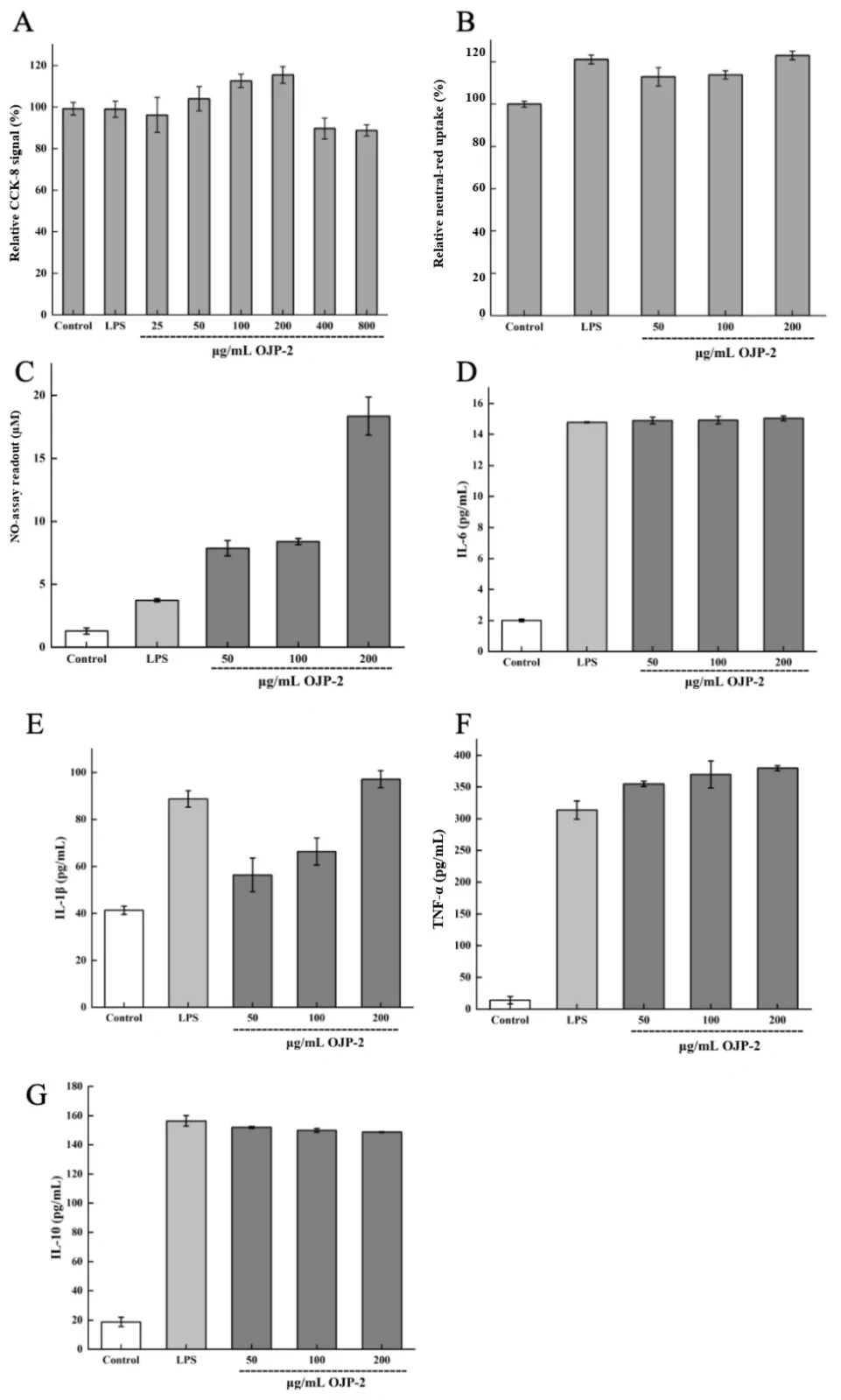
Effects of OJP-2 on basal RAW264.7 cellular responses. (**A**) Relative CCK-8 metabolic signal after 24 h exposure to 25–800 µg/mL OJP-2. (**B**) Relative neutral-red uptake. (**C**) NO-assay readout. (**D**–**G**) IL-6, IL-1β, TNF-α, and IL-10 concentrations after treatment with 50, 100, or 200 µg/mL OJP-2. Bars show mean ± SD. Replicates were technical (six wells for CCK-8 and three measurements for each other basal assay); these counts do not denote independent biological experiments.

The CCK-8 signal was maintained within the 25–200 µg/mL range but declined at 400 and 800 µg/mL (Figure 4A). Therefore, 50–200 µg/mL was selected for subsequent cellular assays.

Neutral-red uptake was higher in the 50, 100, and 200 µg/mL OJP-2 groups than in the control group (Figure 4B). The 50 and 100 µg/mL groups showed similar responses, while the 200 µg/mL group approached the LPS comparison group.

The NO-assay readout increased in all three OJP-2 groups, with the highest value at 200 µg/mL (Figure 4C).

IL-6 concentrations were higher than the control and remained relatively stable across the OJP-2 range (Figure 4D). IL-1β and TNF-α increased with OJP-2 concentration (Figure 4E,F). IL-10 was elevated at all three concentrations but declined slightly from 50 to 200 µg/mL (Figure 4G). The concurrent changes in pro-inflammatory mediators and IL-10 indicate a mixed macrophage activation profile.

Across these basal assays, the response was endpoint-specific rather than uniformly concentration-dependent. The metabolic signal defined a working range in which the lysosomal uptake and NO-related readouts could be evaluated without the decline observed at 400 and 800 µg/mL. Within that range, IL-1β and TNF-α followed an increasing pattern, IL-6 changed little among the three OJP-2 concentrations, and IL-10 remained elevated with a modest downward trend. The combined pattern indicates simultaneous activation and regulatory output in unstimulated macrophages.

### 2.5. OJP-2 Redistributes CD86/CD206 Profiles Under LPS/IFN-γ Conditioning

**Figure 5:**
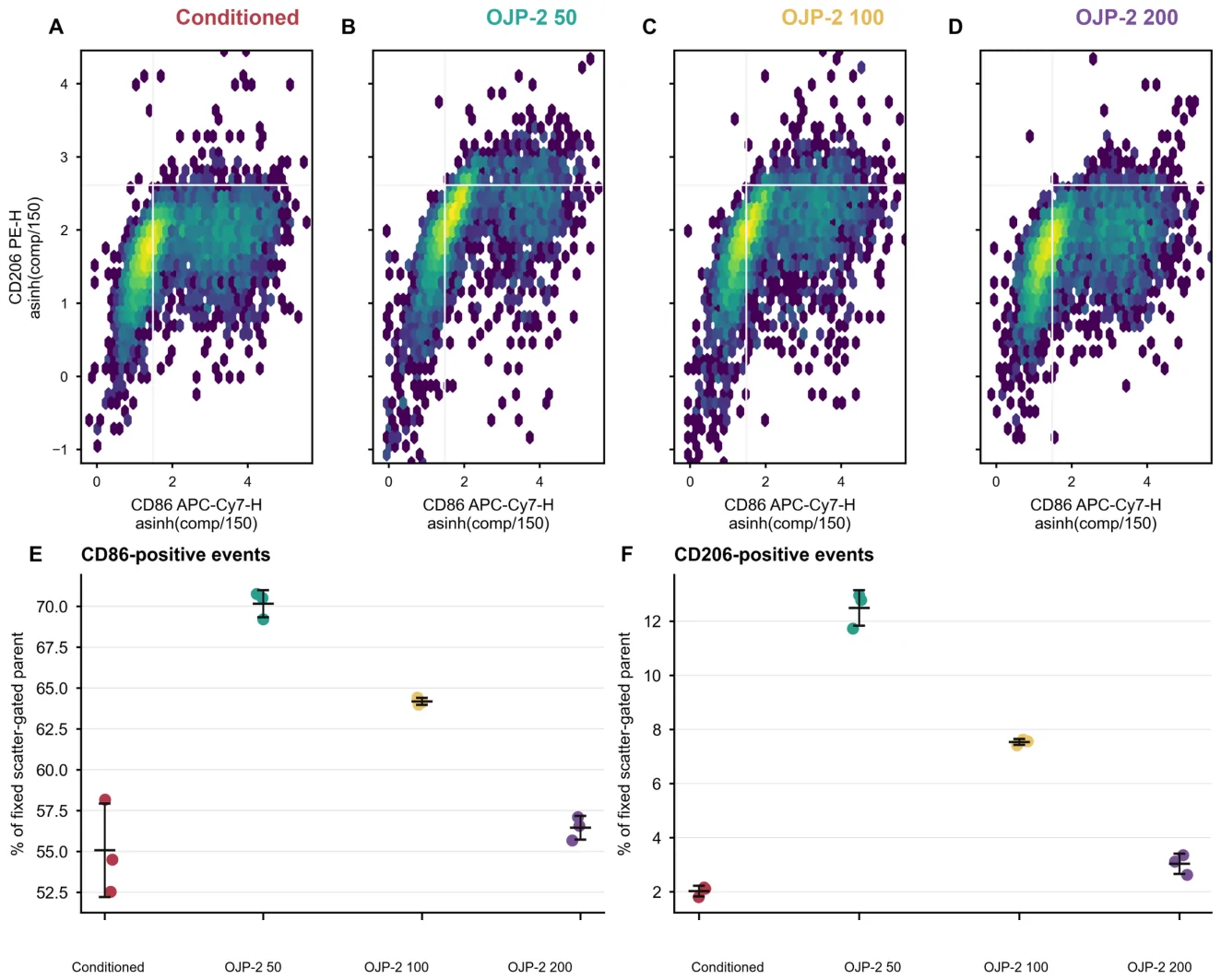
CD86/CD206 marker distributions in LPS/IFN-γ-conditioned RAW264.7 cells. (**A**–**D**) Representative compensated CD86 APC-Cy7-H versus CD206 PE-H density plots for the conditioned control and OJP-2 50, 100, and 200 µg/mL groups. The same FSC-H/SSC-H parent gate and CD86/CD206 thresholds were applied to all samples. (**E**) CD86-positive events (Q2 + Q3). (**F**) CD206-positive events (Q1 + Q2). Points represent individual samples (*n* = 3); horizontal marks and error bars indicate mean ± SD. Group differences were evaluated by Tukey’s HSD test (*p* < 0.05). In panels (**A**–**D**), the color scale from purple through green to yellow indicates increasing local event density. Red, teal, gold, and purple group labels and individual points denote the conditioned control and OJP-2 treatments at 50, 100, and 200 µg/mL, respectively.

The fixed FSC-H/SSC-H parent gate retained 80.34–90.94% of acquired events. CD86-positive events accounted for 55.06 ± 2.87%, 70.16 ± 0.83%, 64.18 ± 0.22%, and 56.45 ± 0.73% of the parent population in the conditioned control, OJP-2 50, OJP-2 100, and OJP-2 200 groups, respectively (Figure 5E). One-way ANOVA yielded *F*_3,8_ = 62.85 and *p* = 6.64 × 10^−6^. Tukey-adjusted comparisons against the conditioned control gave *p* = 1.00 × 10^−5^, 4.04 × 10^−4^, and 0.700 for the 50, 100, and 200 µg/mL groups, respectively.

The corresponding CD206-positive fractions were 2.03 ± 0.20%, 12.49 ± 0.66%, 7.53 ± 0.11%, and 3.03 ± 0.37% (Figure 5F). One-way ANOVA yielded *F*_3,8_ = 439.63 and *p* = 3.24 × 10^−9^; Tukey-adjusted comparisons against the conditioned control gave *p* = 4.26 × 10^−9^, 6.71 × 10^−7^, and 0.0564, respectively. The most pronounced joint redistribution of CD86 and CD206 occurred at 50 µg/mL and gradually diminished at 100 and 200 µg/mL, revealing a concentration-specific, non-monotonic response.

Relative to the conditioned control, the mean CD86-positive fraction was 27.4%, 16.6%, and 2.5% higher at 50, 100, and 200 µg/mL, respectively. The corresponding CD206-positive fractions were approximately 6.15-, 3.71-, and 1.49-fold those of the conditioned control. Both markers therefore showed the same rank order of departure from the conditioned Model, with the strongest redistribution at 50 µg/mL and a return toward the conditioned-control profile as the OJP-2 concentration increased.

### 2.6. OJP-2 Reduces NF-κB-Related Phosphorylation Indices

**Figure 6:**
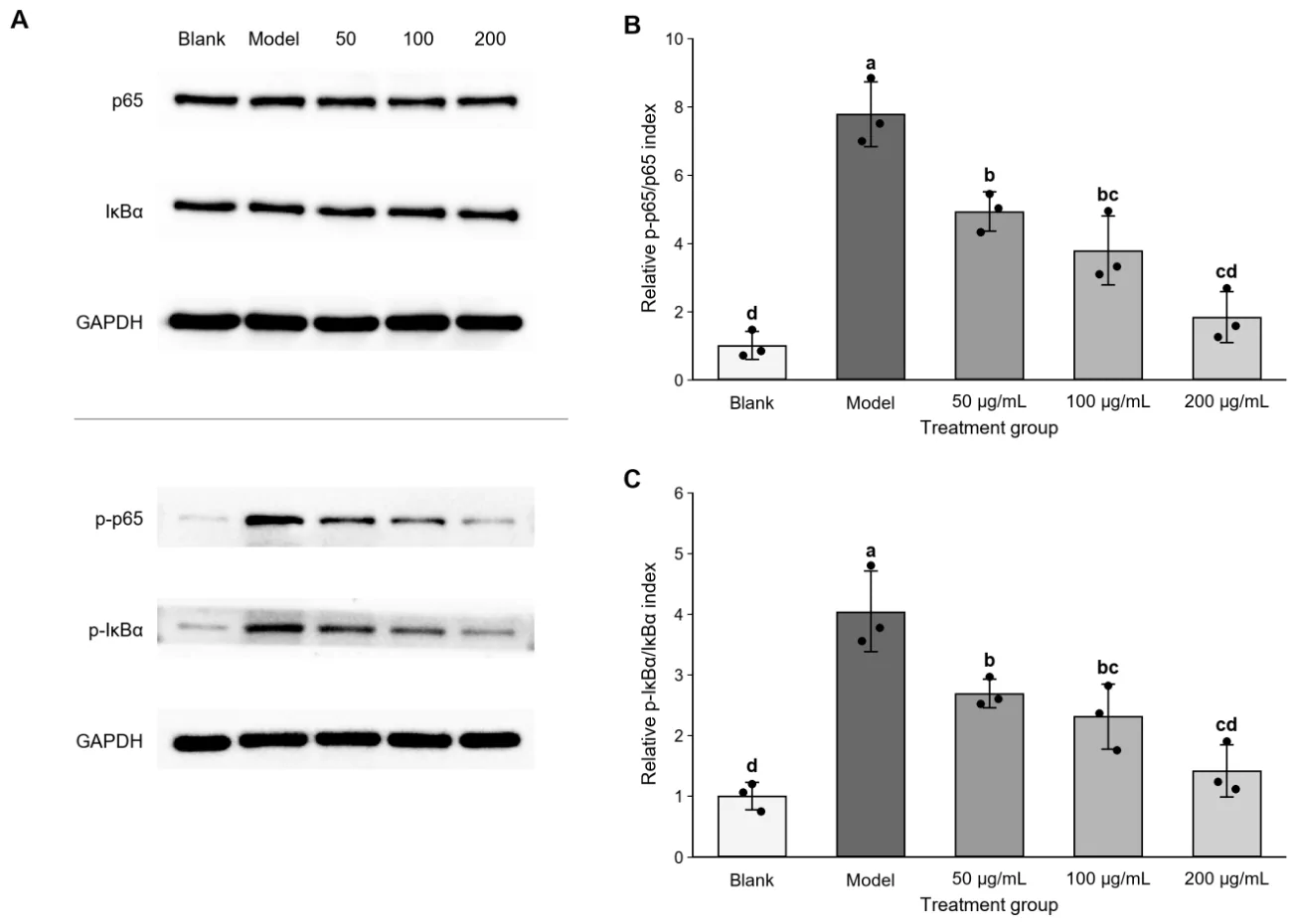
Effects of OJP-2 on NF-κB-related phosphorylation in LPS/IFN-γ-conditioned RAW264.7 cells. (**A**) Representative Western blots. Total p65 and IκBα and their corresponding GAPDH were obtained from one membrane set, while p-p65 and p-IκBα and their corresponding GAPDH were obtained from a second membrane set. (**B**) Relative p-p65/p65 index. (**C**) Relative p-IκBα/IκBα index. Data are shown as mean ± SD with individual values (*n* = 3). Different lowercase letters indicate significant differences using Tukey’s test (*p* < 0.05).

In the Western blot experiment, the relative p-p65/p65 indices for the Blank, Model, and OJP-2 50, 100, and 200 µg/mL groups were 1.00 ± 0.41, 7.77 ± 0.95, 4.93 ± 0.57, 3.77 ± 1.01, and 1.83 ± 0.75, respectively (*n* = 3; Figure 6). One-way ANOVA yielded *p* = 6.32 × 10^−6^. Tukey-adjusted comparisons against the Model gave *p* = 0.00759, 0.000615, and 0.0000207 for the 50, 100, and 200 µg/mL groups, respectively.

The corresponding relative p-IκBα/IκBα indices were 1.00 ± 0.23, 4.04 ± 0.66, 2.69 ± 0.24, 2.31 ± 0.53, and 1.42 ± 0.43. One-way ANOVA yielded *p* = 7.64 × 10^−5^; Tukey-adjusted comparisons against the Model group gave *p* = 0.0282, 0.00584, and 0.000238 for the 50, 100, and 200 µg/mL groups, respectively. Both relative phosphorylation indices declined progressively across the OJP-2 concentration range, and the values at 200 µg/mL approached those of the Blank group.

Compared with the Model mean, the p-p65/p65 index was lower by 36.6%, 51.5%, and 76.4% at 50, 100, and 200 µg/mL, respectively. The p-IκBα/IκBα index was lower by 33.4%, 42.8%, and 64.9%. Unlike the non-monotonic CD86/CD206 profile, both phosphorylation endpoints changed in an ordered concentration-associated manner. The two assay modules therefore captured different components of the conditioned macrophage response: surface-marker redistribution was maximal at the lowest tested concentration, whereas attenuation of NF-κB-related phosphorylation was greatest at the highest concentration.

## 3. Discussion

This study established a continuous preparation-to-response profile for OJP-2. The fraction combined a low apparent molecular mass and narrow HPGPC distribution with a fructose-rich composition and NMR features of an inulin-type fructan. In RAW264.7 cells, OJP-2 enhanced selected functional and secretory readouts under basal conditions, altered CD86/CD206 distributions after LPS/IFN-γ stimulation, and reduced NF-κB-related phosphorylation indices. The direction and concentration pattern varied across endpoints, supporting concentration- and assay-context-dependent macrophage responses.

Papain-assisted simultaneous microwave–ultrasound processing combines enzyme-mediated matrix loosening with rapid energy input and acoustic mixing, an approach aligned with recent ultrasound and enzyme-ultrasound workflows [17,18,19,20,21,22,23]. Across published systems, extraction behavior varies with the source matrix and process conditions, and downstream chromatographic resolution remains central to isolating a defined fraction [19,20,21,22,23,24,25,26,27,28]. In the present workflow, DEAE-52 separated the crude material by ionic interaction and Sephadex G-100 further resolved the selected material by hydrodynamic size. This sequence is important because the composition, NMR features, and cellular responses all refer to chromatographically resolved OJP-2 rather than the crude root extract. Integration of preparation and fractionation with structural and cellular endpoints extends this combined workflow to a low-molecular-weight *O. japonicus* fructan.

The *M*_w_ of 3568 Da places OJP-2 within the low-molecular-weight range relative to many polysaccharide fractions investigated in recent ultrasound-assisted studies [24,25,26]. Its *M*_w_/*M*_n_ value of 1.004 indicates that the detected molecules occupied a particularly narrow hydrodynamic-size range under the HPGPC conditions [30,31]. HPAEC-ED independently established a strongly fructose-enriched composition, with glucose as the only substantial secondary monosaccharide. Agreement between a narrow chromatographic distribution and a focused monosaccharide profile distinguishes OJP-2 from broad, glucose-rich heteropolysaccharide preparations. The compositional value of this comparison lies in the dominant sugar pattern rather than a direct estimate of chain length, because fructose recovery depends on the hydrolysis and detection workflow [32,33]. For compositionally mixed systems, graded-hydrolysis strategies provide an additional route to distinguish component polysaccharides [34].

The NMR evidence connected this compositional signature to a recognizable fructan architecture. Quaternary carbon signals at δ_C_ 103–104 ppm are characteristic of Fruf C-2 positions, whereas the HSQC correlation at δ_H_/δ_C_ 5.24/91.98 ppm supports a terminal α-D-Glcp residue. Long-range contacts from Glcp H-1 and Fruf methylene protons to the Fruf C-2 region are consistent with an α-D-Glcp-(1→2)-β-D-Fruf end group and a chain dominated by β-(2→1)-linked Fruf residues. These features agree with established inulin-type fructan structures [7,8,9,10,11,12,16,35,36]. An additional weak signal class was consistent with possible C-6 substitution of Fruf. Because this NMR assignment was tentative, it only supports the possibility of limited branching and does not establish a specific branch topology or frequency. SEM and AFM complement this molecular description by showing that the lyophilized fraction appeared as porous sheets and heterogeneous granular deposits after drying [37].

The basal-cell assays show that OJP-2 was biologically active within the 50–200 µg/mL working range. Maintenance of the CCK-8 metabolic signal separated this range from the decline observed at higher concentrations [38,39]. Increased neutral-red uptake indicates greater lysosomal dye accumulation [40], while the NO-assay response and cytokine measurements demonstrate altered macrophage secretory activity [41]. Similar activation of macrophage-associated functions has been reported for an *O. japonicus* polysaccharide formulation and for fructans from garlic and *Polygonatum* species [6,15,16]. In OJP-2-treated cultures, the simultaneous elevation of IL-1β, TNF-α, and IL-10, together with a relatively stable IL-6 pattern across the working range, is best described as a mixed pro- and anti-inflammatory mediator profile [13,14,42]. This combination differentiates immunomodulation from a uniformly pro-inflammatory secretory response.

The conditioned-cell experiments revealed a different concentration pattern. The strongest departure from the conditioned control occurred at 50 µg/mL, and both marker-positive fractions moved back toward the conditioned-control profile at 100 and 200 µg/mL. The parallel changes in the separately calculated CD86-positive and CD206-positive fractions fit the current view that macrophage activation occupies a continuum of states rather than a binary M1/M2 switch [43,44]. The data therefore indicate remodeling of the marker distribution within the LPS/IFN-γ-conditioned population. The non-monotonic profile may reflect concentration-dependent engagement of several cellular processes, with the balance among surface-marker states changing across the tested range.

NF-κB-related phosphorylation followed a clearer concentration-associated pattern. Both p-p65/p65 and p-IκBα/IκBα declined progressively across the OJP-2 range, with the largest reductions at 200 µg/mL. Parallel behavior of the two indices is consistent with coordinated modulation of linked NF-κB-related events under inflammatory conditioning. Because the total and phospho-protein targets were quantified from two recorded membrane/image sets, the derived relative indices also incorporate between-set technical variation, despite normalization to the corresponding GAPDH source within each set. This pattern complements the flow-cytometry data: surface-marker redistribution was greatest at 50 µg/mL, but attenuation of intracellular phosphorylation was strongest at 200 µg/mL. Cellular phenotype and signaling intensity therefore did not move as a single linear output.

Viewed together, the basal and conditioned experiments resolve an apparent contrast in the cellular results. OJP-2 enhanced lysosomal, NO-related, and cytokine readouts in unstimulated macrophages, yet moderated NF-κB-related phosphorylation in an LPS/IFN-γ-conditioned state. Fructan responses can depend on molecular size, chain organization, aggregation, concentration, and the activation state of the responding immune cell [8,9,10,13,14]. OJP-2 combines a low molecular mass, a predominantly β-(2→1)-linked fructan chain, a terminal glucosyl residue, and tentative evidence of possible C-6 substitution. These relationships are best treated as a working hypothesis for comparison with structurally distinct fractions, including OJP-1, while the present findings provide a coherent structural basis for continued investigation of O. japonicus fructans and their concentration- and assay-context-dependent macrophage responses. Because endotoxin was not assessed, its potential contribution to the macrophage responses remains unresolved.

## 4. Materials and Methods

### 4.1. Plant Material, Reagents, and Cells

Roots of *O. japonicus* (Thunb.) Ker Gawl. were purchased from Yongjitang (Mianyang, Sichuan, China). Species identification was based on the supplier’s product information and the source and morphological descriptions of *O. japonicus* in the Chinese Pharmacopoeia. The research team did not perform independent botanical authentication, and no voucher specimen was prepared or deposited. Anhydrous ethanol, dichloromethane, n-butanol, hydrogen peroxide, and phenol were obtained from Sinopharm Chemical Reagent Co., Ltd. (Beijing, China). Glucose and Coomassie Brilliant Blue reagent were obtained from Aladdin Biochemical Technology Co., Ltd. (Shanghai, China), and monosaccharide standards were obtained from Sigma (St. Louis, MO, USA). DEAE-52 cellulose and Sephadex G-100 were obtained from Shanghai Yuanye Biotechnology Co., Ltd. (Shanghai, China).

RAW264.7 cells were obtained from the National Certified Cell Culture Collection (Shanghai, China). Dulbecco’s modified Eagle medium (DMEM) and fetal bovine serum (FBS) were obtained from Gibco (New York, NY, USA). Nitric-oxide assay kits were obtained from Nanjing Jiancheng Bioengineering Institute, and ELISA kits were obtained from Beyotime Biotechnology Co., Ltd. (Shanghai, China).

### 4.2. Preparation and Chromatographic Fractionation

The roots were dried to constant mass, milled, and passed through a 20-mesh sieve. Powdered root (10 g) was suspended in 100 mL of water (solid-to-liquid ratio, 1:10, *w*/*v*), and papain (0.20 g; 2% *w*/*w* relative to the dry powder) was added. The suspension was incubated at pH 7.0 and 50 °C for 25 min and then treated simultaneously with microwave irradiation at 208 W and ultrasound at 300 W for 30 min. The extract was deproteinized by the Sevag method, precipitated with ethanol, dialyzed, and lyophilized. The crude-extract yield used for the optimization and validation records was determined gravimetrically from the 80% (*v*/*v*) ethanol-precipitated material before DEAE-52 and Sephadex G-100 purification. The crude-extract yield was calculated as [(m_full − m_empty)/m0] × 100, where m0 is the dry-basis starting material mass, m_empty is the empty-container mass, and m_full is the container plus dried precipitate mass.

A 20 mL portion of crude polysaccharide solution was loaded onto a DEAE-52 cellulose column and sequentially eluted with distilled water, 0.1 M NaCl, and 0.3 M NaCl. Carbohydrate-containing fractions were monitored using the phenol–sulfuric acid method, pooled according to the elution profile, concentrated, desalted, and lyophilized. The first two fractions were separately purified on Sephadex G-100 using water at 0.18 mL/min, yielding OJP-1 and OJP-2. The same OJP-2 preparation was used for physicochemical characterization and cellular assays. Fraction-specific mass records for OJP-1 and OJP-2 were not available; their purification yields are therefore not reported.

### 4.3. UV–Visible and FT-IR Spectroscopy

OJP-2 was scanned from 200 to 400 nm using a UV–visible spectrophotometer (Beijing Puxi General Instrument Co., Ltd., Beijing, China); the spectrum presented in Figure 1D covers 200–300 nm. For FT-IR analysis, 1 mg of lyophilized OJP-2 was mixed with 100 mg KBr and compressed into an approximately 1 mm pellet. Spectra were recorded from 4000 to 400 cm^−1^ with a Nexus IS10 FT-IR spectrometer (Thermo Fisher Scientific, Waltham, MA, USA).

### 4.4. Apparent Molecular-Mass Distribution

The molecular-mass distribution was measured by HPGPC. The calibration plot and OJP-2 chromatographic trace are provided in the Supporting Information. OJP-2 was dissolved in the mobile phase at 5 mg/mL, centrifuged at 12,000 rpm for 10 min, and passed through a 0.22 µm aqueous membrane. Analysis was performed using a Thermo U3000 chromatographic system coupled to a Shimadzu RI-20A refractive-index detector and serial BRT 105-103-101 gel columns (8 mm × 300 mm) (Shimadzu, Tokyo, Japan). The mobile phase was 0.2 M NaCl at 0.7 mL/min, the column temperature was 40 °C, and the injection volume was 50 µL. Molecular-mass parameters were calculated using dextran calibration standards [30,31]. The resulting values are apparent molecular-mass estimates under these calibration and chromatographic conditions. The calibration set comprised P5, P10, P20, P50, P100, P200, P400, and P800 standards (Shodex; stated purities 97–99%), together with a maltose heptasaccharide reference (Yuanye; stated purity 90%). The calibration relationships were linear over the tested range: logMp = −0.2013t + 11.168 (R^2^ = 0.9994), logMw = −0.2011t + 11.158 (R^2^ = 0.9993), and logMn = −0.1998t + 11.107 (R^2^ = 0.9990), where t is retention time in minutes. Dextran-calibrated values depend on hydrodynamic conformation, which may differ between dextran and fructan. The calibration R^2^ values describe goodness of fit rather than sample measurement uncertainty; replicate-based SD or CV estimates were not available (Supporting Information S2).

### 4.5. Monosaccharide Composition

Monosaccharide composition was determined by HPAEC-ED. OJP-2 (5 mg) was hydrolyzed with 2 mL of 3 M trifluoroacetic acid at 80 °C for 2 h. The reported composition is presented as normalized molar fractions obtained under the stated hydrolysis and detection conditions. The hydrolysate was dried under nitrogen, redissolved in 3 mL water, diluted by mixing 50 µL with 950 µL deionized water, centrifuged at 12,000 rpm for 5 min, and filtered through a 0.22 µm membrane. Analysis was performed on a Thermo Fisher ICS5000 system with a Dionex CarboPac PA20 column (150 mm × 3.0 mm) at 30 °C (Thermo Fisher Scientific, Waltham, MA, USA). The flow rate was 0.3 mL/min and the injection volume was 25 µL. The eluents were water, 15 mM NaOH, and 15 mM NaOH containing 100 mM sodium acetate. Peaks were assigned by comparison with monosaccharide standards [32,33]. External-standard peak-area quantification used Csample = Cstd × Asample/Astd, where C and A denote the concentration and peak area, respectively. Mass concentrations were converted to molar amounts and normalized to obtain the reported molar fractions. Formal fructose-recovery validation, including spike recovery, was not performed.

### 4.6. Scanning Electron and Atomic-Force Microscopy

For scanning electron microscopy (SEM), approximately 1 mg of dried OJP-2 was mounted, sputter-coated with gold for 15 min, and examined with an S-4800 microscope (Hitachi, Tokyo, Japan) at 250× and 3000×. The displayed fields recorded 10.0 kV in SE(M) mode and working distances of 12.1 and 11.9 mm, respectively.

For atomic-force microscopy (AFM), OJP-2 was dissolved in deionized water at 5 mg/mL, and 5 µL was deposited on mica and dried before imaging with a scanning-probe microscope from Aijian Technology Instruments Co., Ltd. (Shanghai, China). The displayed AFM field was 10 µm × 10 µm, and the prepared-state topography was analyzed as described previously [37].

### 4.7. NMR Spectroscopy

OJP-2 (70 mg) was exchanged with D_2_O and analyzed in a 5 mm NMR tube using an Agilent DD2 500 NMR spectrometer (Agilent, Santa Clara, CA, USA) equipped with a broadband room-temperature probe. Spectra were acquired at 23 °C with observed frequencies of 499.77 MHz for ^1^H and 125.68 MHz for ^13^C. One-dimensional ^1^H and ^13^C spectra and two-dimensional gCOSY, gHSQC, gHMBC, NOESY, and TOCSY spectra were collected. The NOESY and TOCSY mixing times were 300 and 500 ms, respectively; gHSQC was optimized for a one-bond coupling of 146 Hz and gHMBC for a long-range coupling of 8 Hz. The numbers of indirect-dimension increments were 128, 96, 200, 200, and 200 for gCOSY, gHSQC, gHMBC, NOESY, and TOCSY, respectively. The ^13^C spectrum comprised 1608 transients. Chemical shifts were expressed in ppm, and assignments were made from the combined one- and two-dimensional spectra with reference to published fructan structures [35,36].

### 4.8. Basal RAW264.7 Cell Assays

Lyophilized OJP-2 from the same preparation used for physicochemical characterization was dispersed in distilled water and passed through a sterile membrane filter before cell treatment. The OJP-2 preparation was not tested for endotoxin.

RAW264.7 cells were maintained in DMEM containing 10% FBS and 1% penicillin-streptomycin at 37 °C in 5% CO_2_. For the CCK-8 assay, cells were seeded in 96-well plates at 1 × 10^5^ cells/well and allowed to attach for 24 h. The medium was replaced with complete medium containing OJP-2 at 0, 25, 50, 100, 200, 400, or 800 µg/mL for 24 h. Six technical replicate wells were used per group. CCK-8 reagent (10 µL/well) was added for 2 h, and absorbance was measured at 450 nm. The relative metabolic signal was expressed against the control group.

For neutral-red uptake, exposed cells were washed with phosphate-buffered saline (PBS), incubated with 0.5% neutral-red solution for 30 min, and washed again. Cell-associated dye was released with 100 µL lysis solution for 10 min, and absorbance was measured at 550 nm.

An NO-assay readout was obtained from culture supernatants using a commercial kit, with absorbance measured at 540 nm. For cytokine measurements, 1 × 10^5^ RAW264.7 cells were seeded in 24-well plates for 24 h and then exposed to OJP-2 (50, 100, or 200 µg/mL) or LPS (1 µg/mL) for a further 24 h. IL-1β, IL-6, IL-10, and TNF-α were measured in culture supernatants by ELISA, with absorbance recorded at 450 nm. Neutral-red, NO, and ELISA results each comprised three technical replicate measurements per group. The retained records do not establish a separate count of independent biological experiments for these basal assays.

The CCK-8 signal was used as an indicator of cellular metabolic activity [38,39], neutral-red uptake as an indicator of lysosomal uptake [40], and nitrite-based colorimetry as an NO-related readout [41].

### 4.9. Flow Cytometry

For flow cytometry, RAW264.7 cells were stimulated with LPS (100 ng/mL) and IFN-γ (20 ng/mL) for 48 h to generate LPS/IFN-γ-conditioned macrophages. The treatment groups then received OJP-2 at 50, 100, or 200 µg/mL for 24 h, representing low-, medium-, and high-concentration treatments, respectively. Three independent samples were analyzed per group, and comparisons were made among the conditioned control and the OJP-2 50, 100, and 200 µg/mL groups.

Cells were stained with CD86-APC/Cy7 (BioLegend, San Diego, CA, USA, clone GL-1, supplied identifier B423660; 5 µL/test) and CD206-PE (Elabscience, Houston, TX, USA, supplied identifier WE044T6JZ9714; 5 µL/test). CD86 and CD206 distributions were used to evaluate changes in macrophage surface-marker profiles.

FCS3.1 files were reanalyzed using FlowIO 1.4.0. The available single-stained reference files and the embedded 12 × 12 spillover matrix were retained with the source data for the fluorescence-compensation workflow. The embedded matrix was applied to all fluorescence channels. A sample-independent parent gate was fitted in log10-transformed FSC-H/SSC-H space using equal event contributions from all files, and the central 85% pooled covariance ellipse was applied unchanged to each sample. CD86 was evaluated in the compensated APC-Cy7 channel and CD206 in the compensated PE channel. Fixed thresholds of 314.64 and 1017.42 compensated linear units were used for CD86 and CD206, respectively. CD86 positivity was calculated as Q2 + Q3 and CD206 positivity as Q1 + Q2. The single-stained reference files were 1.17_M1-CD86_1.fcs and 1.17_M1-CD206_2.fcs. Additional unstained, FMO, isotype, viability, and singlet-gating records were not available.

### 4.10. Western Blot Analysis

For Western blot analysis, RAW264.7 cells were assigned to Blank, Model, and Model-plus-OJP-2 groups. Three independent experiments were performed for each group. The Model group was exposed to LPS (100 ng/mL) and IFN-γ (20 ng/mL; MedChemExpress, Monmouth Junction, NJ, USA, supplied identifier 669167) for 24 h. The treatment groups subsequently received OJP-2 at 50, 100, or 200 µg/mL for a further 24 h.

Cells were washed twice with PBS and lysed on ice for 30 min in RIPA buffer supplemented with PMSF, protease-inhibitor cocktail, and phosphatase inhibitors. Lysates were centrifuged at 12,000 rpm for 10 min at 4 °C. Protein concentrations were determined by BCA assay and adjusted to 2 µg/µL. Samples were mixed with 5× reducing loading buffer at 4:1 (*v*/*v*) and heated at 100 °C for 10 min. Twenty micrograms of protein per lane were separated by SDS-PAGE (80 V for 20 min through the stacking gel and 120 V for approximately 1 h through the resolving gel) and transferred to PVDF membranes for 45 min. Membranes were blocked for 10 min with rapid protein-free blocking solution and incubated at 4 °C overnight with antibodies against NF-κB p65 (Affinity Biosciences, Cincinnati, OH, USA, AF5006; 1:2000), phospho-NF-κB p65 Ser536 (ImmunoWay, San Jose, CA, USA, YM8442; 1:2000), IκBα (ABclonal, Woburn, MA, USA, A19714; 1:2000), phospho-IκBα Ser36 (ABclonal, AP0999; 1:2000), or GAPDH (Servicebio, Wuhan, China, GB15004-100; 1:3000). After three 10 min TBST washes, membranes were incubated for 60 min with HRP-conjugated goat anti-rabbit IgG (Servicebio, GB23303; 1:10,000), washed again, and developed by ECL using an SCG-W3000 chemiluminescence imaging system (Servicebio). Band intensities were quantified using ImageJ 1.54.

The total-protein and phospho-protein targets originated from two recorded membrane/image sets, each with their corresponding GAPDH source. Within each set, target-band intensity was normalized to the corresponding GAPDH signal. The GAPDH-normalized phospho-protein signal was then divided by the GAPDH-normalized total-protein signal, and the resulting relative phosphorylation index was expressed relative to the Blank-group mean. Thus, the reported endpoints are relative p-p65/p65 and p-IκBα/IκBα indices rather than absolute phosphorylation stoichiometry. Representative source images and the normalized densitometry data are provided in Supporting Information S5–S6 (Table S4 and Figures S4 and S5).

### 4.11. Statistical Analysis

Data are presented as mean ± SD. Basal cellular assays were summarized descriptively. Basal-assay means and SDs summarize technical replicates; independent samples or experiments were the units of analysis for flow cytometry and Western blotting, respectively [45]. Normality was assessed by Shapiro–Wilk tests for each analyzed group and endpoint; the test statistics and *p* values are provided in Supporting Information S4.

For flow cytometry, CD86+ and CD206+ percentages were analyzed separately by one-way ANOVA followed by Tukey’s HSD test. Three independent samples were included per group, and individual values, means, and SDs were reported.

For Western blot analysis, relative p-p65/p65 and p-IκBα/IκBα indices were analyzed separately by one-way ANOVA followed by Tukey’s multiple-comparison test. Three independent experiments were included per group, and values are shown as mean ± SD with individual values overlaid. Differences were considered significant at *p* < 0.05.

## 5. Conclusions

OJP-2 is a low-molecular-weight, fructose-rich inulin-type fructan characterized by a predominantly β-(2→1)-linked Fruf chain and a terminal α-D-Glcp residue, with a weak signal class tentatively assigned to possible C-6 substitution. OJP-2 modulated basal RAW264.7 cellular and cytokine responses, produced concentration-specific redistribution of CD86/CD206 profiles under LPS/IFN-γ conditioning, and reduced NF-κB-related phosphorylation indices. These findings support OJP-2 as a candidate fructan for further investigation of immunomodulatory activity.

## Figures and Tables

**Table 1:** Proposed NMR assignments for OJP-2. Open-ended linkage notation denotes continuation of the polysaccharide chain.

Code	Proposed Residue	Characteristic NMR Signals	Structural Assignment
A	→1)-β-D-Fruf-(2→	C-2 cluster at δ_C_ 103.08–103.87 ppm; HMBC correlations at δ_H_ 3.5–3.8/δ_C_ ~103 ppm	Predominant β-(2→1)-linked Fruf sequence
B	Terminal β-D-Fruf-(2→	Terminal Fruf signals within the C-2 and methylene regions	Terminal Fruf residue
C	→1,6)-β-D-Fruf-(2→	Downfield methylene signal and associated HMBC features	Possible C-6 substitution of Fruf (tentative NMR assignment)
D	Terminal α-D-Glcp-(1→	HSQC at δ_H_/δ_C_ 5.24/91.98 ppm; HMBC near δ_H_/δ_C_ 5.24/~103 ppm	Terminal α-D-Glcp linked to the Fruf chain

## Data Availability

The data supporting the findings of this study are available from the corresponding author upon reasonable request.

## Supplementary Materials

The following supporting information can be downloaded at https://www.mdpi.com/article/10.3390/molecules31183194/s1. Figure S1. Representative class-level motif of an inulin-type fructan containing a terminal α-D-Glcp residue and predominantly β-(2→1)-linked D-Fruf residues. Figure S2. HPGPC calibration relationships for Mp, Mw, and Mn. Figure S3. Enlarged ^1^H (left) and ^13^C (right) NMR regions supporting the principal assignments. Figure S4. Representative full-frame Western blot source images from experiment 1: phospho-protein membrane set. Figure S5. Representative full-frame Western blot source images from experiment 1: total-protein membrane set. Table S1. HPGPC calibration equations; t denotes retention time in minutes. Table S2. NMR assignments for OJP-2. Table S3. Shapiro–Wilk tests for flow cytometry and Western blot endpoints. Table S4. Normalized Western blot densitometry data from three independent experiments. Table S5. Crude-extract yields from five independent extractions.
