# Supplementary material for: Structural Analysis of an Inulin-Type Fructan from Ophiopogon japonicus and Its Immunomodulatory Properties
Source: Molecules. 2026 Sep 10;31(18):3194. doi: 10.3390/molecules31183194 (PMC13609086; doi:10.3390/molecules31183194)
Supplement: Supplementary file 1 [file molecules-31-03194-s001.zip › molecules-4532509-supplementary.pdf]

Supporting Information

Structural Analysis of an Inulin-Type Fructan from Ophiopogon japonicus and Its Immunomodulatory Properties

S1. Representative fructan motif

Representative class-level motif showing a terminal α-D-Glcp residue linked to a predominantly β-(2→1)-linked D-Fruf sequence. The motif illustrates the residue and linkage nomenclature applied in the manuscript.

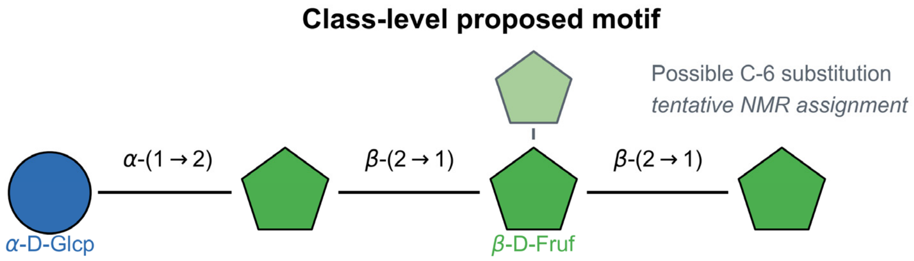

Figure S1. Representative class-level motif of an inulin-type fructan containing a terminal α-D-Glcp residue and predominantly β-(2→1)-linked D-Fruf residues.

S2. HPGPC calibration information

The calibration set comprised P5, P10, P20, P50, P100, P200, P400, and P800 standards (Shodex; stated purities 97–99%), together with a maltose heptasaccharide reference (Yuanye; stated purity 90%).

Table S1. HPGPC calibration equations; t denotes retention time in minutes.

Parameter	Calibration equation	R^2
log Mp	−0.2013t + 11.168	0.9994
log Mw	−0.2011t + 11.158	0.9993
log Mn	−0.1998t + 11.107	0.9990

OJP-2: Mp = 3588 Da, Mw = 3568 Da, Mn = 3553 Da, and Mw/Mn = 1.004. These are apparent molecular masses based on hydrodynamic size relative to dextran calibrants. Differences between dextran and fructan conformation can affect the estimates. The R^2 values characterize calibration fit and are not uncertainty intervals for OJP-2. Independent analytical replicate records were not available to derive SD, CV, or confidence intervals.

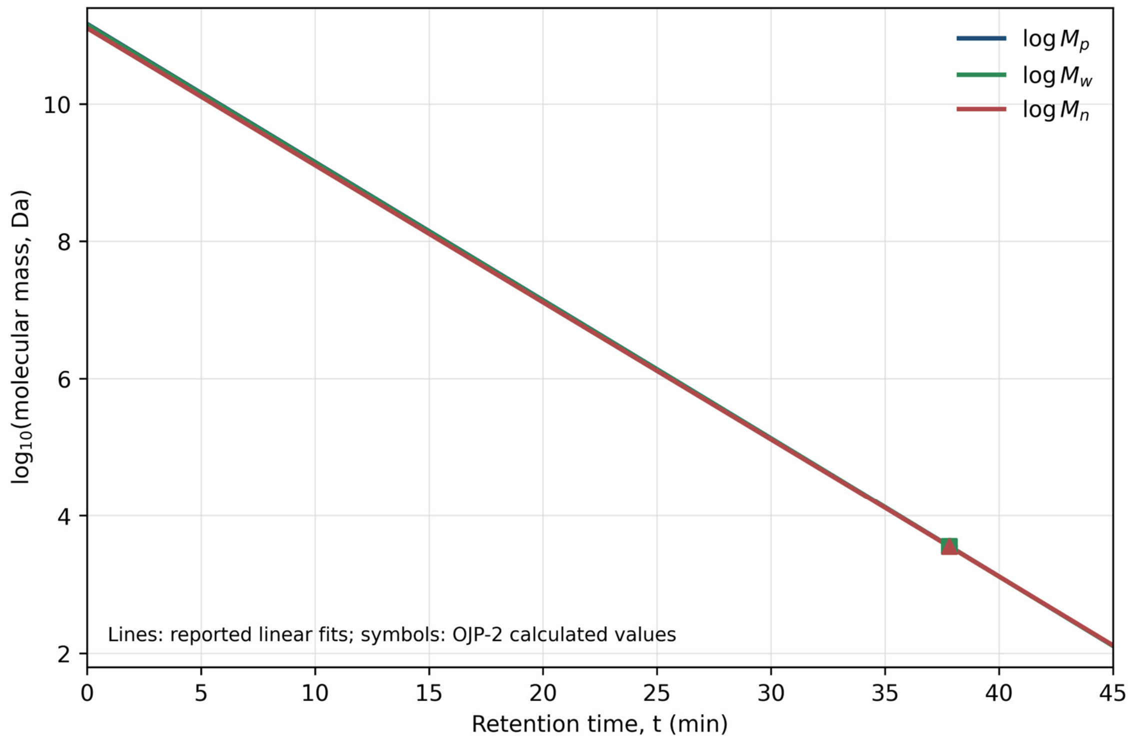

Figure S2. HPGPC calibration relationships for Mp, Mw, and Mn.

S3. NMR peak-assignment table

Table S2. NMR assignments for OJP-2.

Label	Residue	H1/C1	H2/C2	H3/C3	H4/C4	H5/C5	H6/C6
A	β-1,2-Fru-(2→	3.66(3.78)/62.15	–/103.03	4.07/76.87	3.93/74.23	3.70/81.07	3.60(3.72)/61.26
B	β-t-Fru-(2→	3.53(3.72)/60.47	–/103.88	4.05/76.87	3.89/74.58	3.78/79.55	3.63(3.86)/60.47
C	β-1,2,6-Fru-(2→	3.58(3.77)/63.30	–/103.56	4.01/74.23	3.87/75.01	3.79/80.98	3.65(3.81)/62.98
D	α-t-Glc-(1→	5.25/91.90	3.38/70.70	3.69/74.23	3.30/69.17	3.87/73.32	3.66(3.72)/60.20

The assignment at approximately δH/δC 5.25/91.90 ppm supports a terminal α-D-Glcp residue. The major ^{13}C signals near 103–104 ppm are consistent with Fruf C-2 environments. The C-6-related signal class is retained as a tentative assignment.

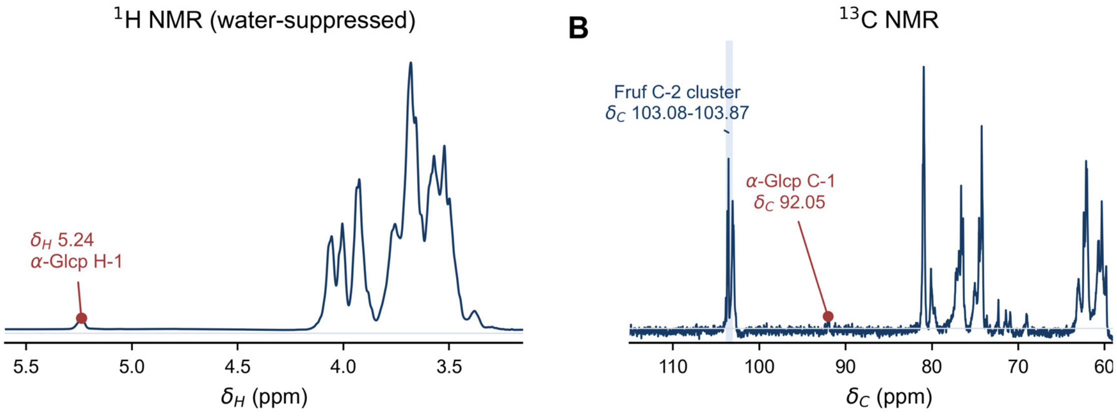

Figure S3. Enlarged ¹H (left) and ¹³C (right) NMR regions supporting the principal assignments.

S4. Normality tests

Shapiro–Wilk tests were applied within each flow-cytometry and Western blot group and endpoint (n = 3 independent samples or experiments per group). All P values were greater than 0.05.

Table S3. Shapiro–Wilk tests for flow-cytometry and Western blot endpoints.

Analysis	Endpoint	Group	n	W	P
Flow	CD86+ (%)	LPS/IFN-γ-conditioned	3	0.9706	0.6708
Flow	CD86+ (%)	OJP-2 50 μg/mL	3	0.8613	0.2711
Flow	CD86+ (%)	OJP-2 100 μg/mL	3	0.9637	0.6340
Flow	CD86+ (%)	OJP-2 200 μg/mL	3	0.9787	0.7205
Flow	CD206+ (%)	LPS/IFN-γ-conditioned	3	0.8398	0.2136
Flow	CD206+ (%)	OJP-2 50 μg/mL	3	0.8575	0.2606
Flow	CD206+ (%)	OJP-2 100 μg/mL	3	0.9528	0.5817
Flow	CD206+ (%)	OJP-2 200 μg/mL	3	0.9625	0.6280
WB	p-p65/p65	Blank	3	0.8754	0.3110
WB	p-p65/p65	Model	3	0.9380	0.5194
WB	p-p65/p65	OJP-2 50 μg/mL	3	0.9791	0.7229
WB	p-p65/p65	OJP-2 100 μg/mL	3	0.8420	0.2193
WB	p-p65/p65	OJP-2 200 μg/mL	3	0.9105	0.4199
WB	p-IκBα/IκBα	Blank	3	0.9560	0.5962
WB	p-IκBα/IκBα	Model	3	0.8777	0.3178
WB	p-IκBα/IκBα	OJP-2 50 μg/mL	3	0.8897	0.3536
WB	p-IκBα/IκBα	OJP-2 100 μg/mL	3	0.9924	0.8337
WB	p-IκBα/IκBα	OJP-2 200 μg/mL	3	0.8602	0.2680

S5. Western blot normalization

Total and phospho-protein bands were normalized to their corresponding GAPDH source within each recorded membrane/image set. The resulting relative p-p65/p65 and p-IκBα/IκBα indices were expressed relative to the Blank-group mean. Three independent experiments were performed, each with separate phospho-protein and total-protein membrane sets. Table S4 presents the normalized values used in Figure 6B,C. Figures S4-S5 show representative source images from the first independent experiment.

For each experiment, the GAPDH-normalized phospho-protein signal was divided by the corresponding GAPDH-normalized total-protein signal. These ratios were then normalized to the mean ratio of the three Blank samples. The group order is Blank, Model, and OJP-2 at 50, 100, and 200 μg/mL.

Table S4. Normalized Western blot densitometry data from three independent experiments.

Endpoint	Group	Experiment 1	Experiment 2	Experiment 3
p-p65/p65	Blank	0.703332	1.461909	0.834760
p-p65/p65	Model	6.989955	8.829504	7.500156
p-p65/p65	OJP-2 50 μg/mL	4.312321	5.443904	5.021302
p-p65/p65	OJP-2 100 μg/mL	3.081754	4.930107	3.312512
p-p65/p65	OJP-2 200 μg/mL	1.244791	2.678113	1.572383
p-IκBα/IκBα	Blank	1.054959	1.194274	0.750767
p-IκBα/IκBα	Model	3.550268	4.792933	3.769936
p-IκBα/IκBα	OJP-2 50 μg/mL	2.513716	2.961698	2.601133
p-IκBα/IκBα	OJP-2 100 μg/mL	2.362384	2.812313	1.752146
p-IκBα/IκBα	OJP-2 200 μg/mL	1.230657	1.904565	1.110962

Values are relative phosphorylation indices normalized to the Blank-group mean and are shown to six decimal places.

S6. Western blot source images

Repeat 1 - phospho-protein set

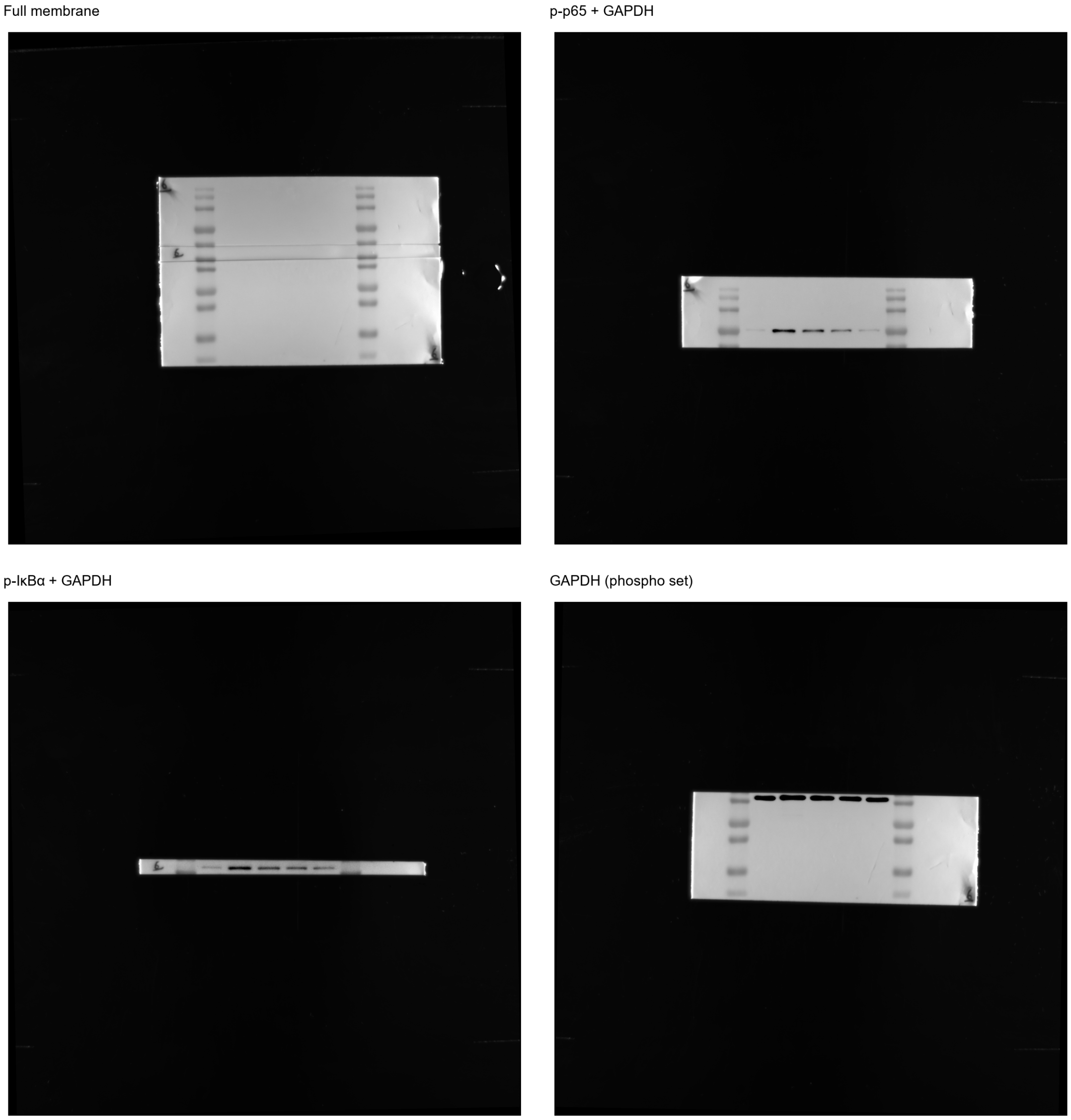

Figure S4. Representative full-frame Western blot source images from experiment 1: phospho-protein membrane set. The panels show the full-membrane bright-field reference and the corresponding target-band images. Target-band images are bright-field/chemiluminescence composites of membrane strips. Source images are shown with their complete frames. The sample order is Blank, Model, and OJP-2 at 50, 100, and 200 μg/mL.

Repeat 1 - total-protein set

Full membrane

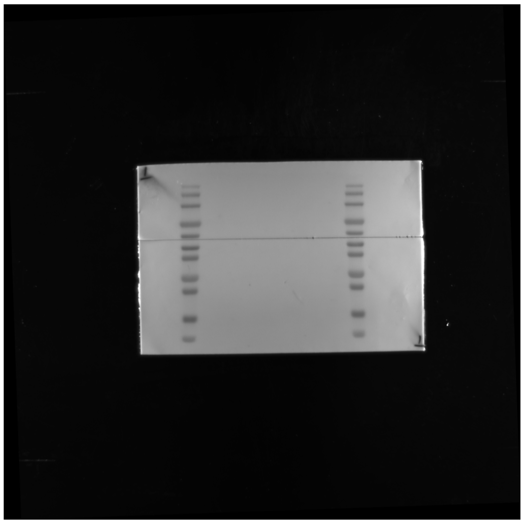

p65 + GAPDH

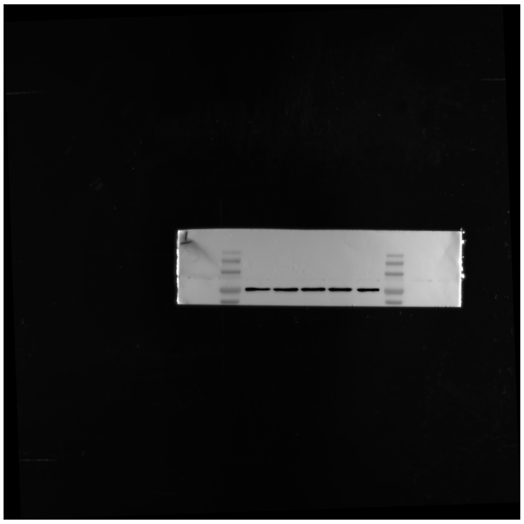

IκBα + GAPDH

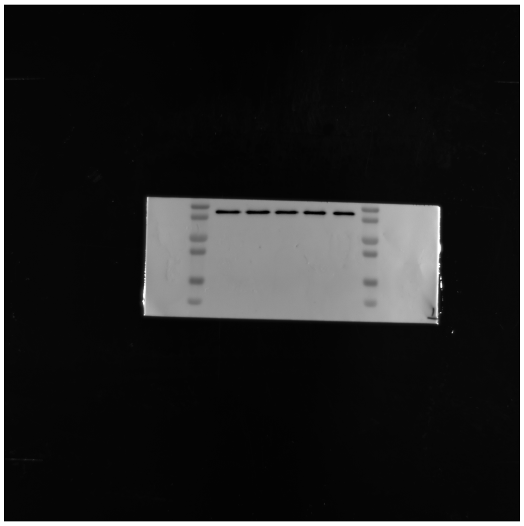

GAPDH (total set)

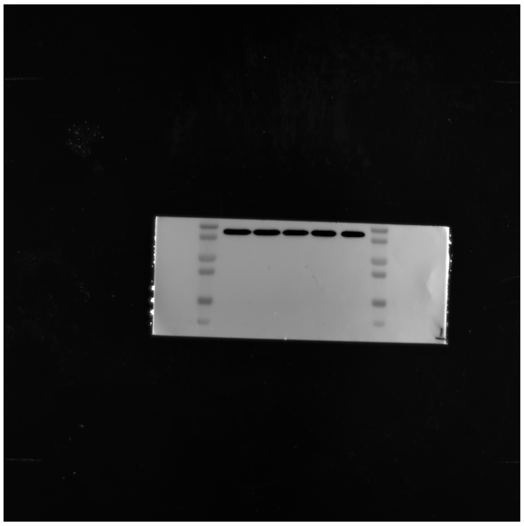

Figure S5. Representative full-frame Western blot source images from experiment 1: total-protein membrane set. The panels show the full-membrane bright-field reference and the corresponding target-band images. Target-band images are bright-field/chemiluminescence composites of membrane strips. Source images are shown with their complete frames. The sample order is Blank, Model, and OJP-2 at 50, 100, and 200 μg/mL.

S7. Crude-extract yields

Five independent extractions were performed at 208 W microwave power, 300 W ultrasound power, 25 min enzymatic treatment, and 30 min extraction. The reported values are gravimetric yields of the dried 80% (v/v) ethanol precipitate before DEAE-52 and Sephadex G-100 purification, without correction for total sugar purity.

Table S5. Crude-extract yields from five independent extractions.

Independent extraction	Crude-extract yield (%)
1	43.67
2	43.39
3	43.77
4	43.81
5	43.20
Mean ± SD (n = 5)	43.568 ± 0.263

Yield (%) = [(m_full − m_empty)/m0] × 100, where m0 is the dry-basis starting-material mass, m_empty is the empty-container mass, and m_full is the container plus dried precipitate mass. The coefficient of variation was 0.604%. Fraction-specific mass records for OJP-1 and OJP-2 were not available, so chromatographic purification yields are not reported.
